# Preterm Birth Following Active Surveillance vs Loop Excision for Cervical Intraepithelial Neoplasia Grade 2

**DOI:** 10.1001/jamanetworkopen.2024.2309

**Published:** 2024-03-14

**Authors:** Kathrine Dyhr Lycke, Johnny Kahlert, Dina Overgaard Eriksen, Camilla Omann, Lars Henning Pedersen, Iben Sundtoft, Rebecca Landy, Lone Kjeld Petersen, Anne Hammer

**Affiliations:** 1 Department of Obstetrics and Gynecology, Gødstrup Hospital, Herning, Denmark; 2 NIDO, Centre for Research and Education, Gødstrup Hospital, Herning, Denmark; 3 Department of Clinical Medicine, Aarhus University, Aarhus, Denmark; 4 Department of Clinical Epidemiology, Aarhus University Hospital, Aarhus, Denmark; 5 Department of Obstetrics and Gynecology, Aarhus University Hospital, Aarhus, Denmark; 6 Division of Cancer Epidemiology and Genetics, National Cancer Institute, National Institutes of Health, Department of Health and Human Services, Bethesda, Maryland; 7 Department of Obstetrics and Gynecology, Odense University Hospital, Odense, Denmark; 8 Department of Clinical Research, University of Southern Denmark, Odense, Denmark; 9 Department of Biomedicine, Aarhus University, Aarhus, Denmark

## Abstract

**Importance:**

Active surveillance for cervical intraepithelial neoplasia grade 2 (CIN2) is being implemented in many high-income countries due to the association of excisional treatment with preterm birth. However, it is unknown whether active surveillance results in lower risk of preterm birth given that cervical dysplasia itself is associated with higher risk of preterm birth.

**Objective:**

To compare the preterm birth risk between women with CIN2 undergoing active surveillance or immediate loop electrosurgical excision procedure (LEEP).

**Design, Setting, and Participants:**

This historical population-based cohort study included women with a first-time diagnosis of CIN2 and a subsequent singleton birth from 1998 to 2018 in Denmark. Women with prior CIN grade 3 or greater or LEEP were excluded. Data were collected from 4 Danish health care registries. Analyses were conducted from October 2022 to June 2023.

**Exposure:**

Women were categorized into active surveillance (cervical biopsy and/or cytology) or immediate LEEP based on their first cervical sample after CIN2 diagnosis. The active surveillance group was further subdivided based on whether a delayed LEEP was performed within 28 months from CIN2 diagnosis.

**Main Outcomes and Measures:**

Risk of preterm birth (<37 + 0 weeks) was assessed and relative risks (RRs) were calculated using modified Poisson regression. Analyses used inverse probability treatment weighting of the propensity scores to adjust for age, parity, calendar year, index cytology, and smoking.

**Results:**

A total of 10 537 women with CIN2 and a singleton birth were identified; 4430 (42%) underwent active surveillance and 6107 (58%) were treated with immediate LEEP. For both groups, most were aged 23 to 29 years at CIN2 diagnosis (3125 [70%] and 3907 [64%], respectively). Overall, 869 births (8.2%) were preterm. The risk of preterm birth was comparable between active surveillance and immediate LEEP (RR, 1.03; 95% CI, 0.90-1.18). However, for women undergoing delayed LEEP after active surveillance (1539 of the active surveillance group [35%]), the risk of preterm birth was higher than for women treated with immediate LEEP (RR, 1.29; 95% CI, 1.08-1.55).

**Conclusions and relevance:**

In this cohort study of women with CIN2, the risk of preterm birth was comparable between active surveillance and immediate LEEP. However, delayed LEEP was associated with 30% higher risk of preterm birth than immediate LEEP. Thus, risk stratification at CIN2 diagnosis is important to identify women with increased risk of delayed LEEP.

## Key Points

**Question:**

Is active surveillance for cervical intraepithelial neoplasia grade 2 (CIN2) associated with reduced risk of preterm birth compared with excisional treatment?

**Findings:**

In this cohort study of 10 537 women with CIN2, the risk of preterm birth was comparable between active surveillance and immediate loop electrosurgical excision procedure (LEEP) for CIN2, while delayed LEEP after active surveillance was associated with a significantly increased risk of preterm birth.

**Meaning:**

In this study, active surveillance was not associated with lower risk of preterm birth; however, given that delayed LEEP was associated with increased risk, risk stratification at CIN2 diagnosis is important to reduce the risk of preterm birth.

## Teaser

This cohort study compares the risk of preterm birth between women with cervical intraepithelial neoplasia grade 2 (CIN2) who underwent active surveillance or an immediate loop electrosurgical excision procedure.

## Introduction

Cervical intraepithelial neoplasia grade 2 (CIN2), a high-grade precursor lesion, has historically been treated with surgical excision, which efficiently reduces the risk of progression to cervical cancer. Yet, studies have shown that 50% to 60% of CIN2 cases spontaneously regress within 2 years.^1,2,3^ This implies risk of overtreatment if all women with CIN2 are treated surgically. Furthermore, surgical treatment is associated with an increased risk of preterm birth and preterm premature rupture of membranes (PPROM) compared with the general population.^4,5,6,7^ Thus, to benefit from the high regression rates and reduce the harms associated with surgical excision, many high-income countries have implemented active surveillance for CIN2 as an option in younger women.^8^ Instead of surgical treatment, women undergo regular follow-up visits for 2 years with surgical treatment in the case of progression to CIN grade 3 or greater (CIN3+) or persistent CIN2 after 2 years.^8^

However, this conservative approach to CIN2, in which the lesion is left untreated, has been introduced without considering that women with untreated CIN have a higher risk of preterm birth compared with the general population.^6,9,10,11,12,13,14^ Therefore, the anticipated reduction in the number of preterm births following the introduction of active surveillance for CIN2 may be limited. In this study, we estimate the risk of preterm birth in women with CIN2 and compare the risk between women who underwent active surveillance or immediate surgical treatment.

## Methods

### Setting

We conducted a historical nationwide population-based cohort study using data from Danish health care registries. Approval from the ethics committee is not required for registry-based research according to Danish legislation. The study was reported to the Danish Data Protection Agency through registration at Aarhus University, Central Region Denmark, and the Danish Health Data Authority. This study followed the Strengthening the Reporting of Observational Studies in Epidemiology (STROBE) reporting guideline for cohort studies.

In Denmark, each resident is assigned a personal identification number at birth or upon immigration, which enables accurate linkage of the Danish registries.^15^ We obtained data from 4 registries: (1) the Danish Civil Registration System, which holds information on age, residence, migration, and vital status^15^; (2) the Danish Pathology Registry, which contains information on cytologic and histologic samples collected in public and private clinics and hospitals^16^; (3) the Danish Medical Birth Registry, which contains information on all births from gestational week 22^17^; and (4) the Danish National Patient Registry, which holds diagnoses and procedures from all private and public inpatient and outpatient hospital contacts.^18^

In Denmark, residents have equal and free of charge access to the health care system, including screening. In 1986, the first national screening guidelines were published recommending triennial cytology-based screening for women aged 23 to 59 years. Women with an abnormal screening test may be referred for colposcopy with collection of cervical biopsies. The biopsy and associated cytology result determine the subsequent clinical management. For women with CIN2, there are 2 options: (1) active surveillance for all women of fertile age, regardless of family planning, associated cytology, lesion size, and other factors, or (2) immediate surgical excision with loop electrosurgical excision procedure (LEEP). Active surveillance was regionally implemented in Denmark in 1995 and nationally in 2013.^19^ It consists of regular follow-up visits with colposcopy, cytology, and cervical biopsies. In the case of progression to CIN3 or persistent disease after 2 years, a delayed LEEP is recommended.

### Study Cohort

We used the Danish Pathology Registry and Danish Medical Birth Registry^16,17^ to identify our study cohort: women aged 18 to 40 years with a first-time diagnosis of CIN2 on cervical biopsies between January 1, 1998, and December 31, 2018, who had a singleton birth after their CIN2 diagnosis (eFigure in Supplement 1). Women with a prior diagnosis of CIN3+ or LEEP were excluded.

### Exposure

We used the Danish Pathology Registry to determine whether women had undergone active surveillance or immediate LEEP. If the first recorded sample within 10 months after CIN2 was a cervical biopsy and/or cytology, we classified the woman as having undergone active surveillance. Women with a first subsequent record of LEEP were categorized as undergoing immediate LEEP. Women with no record of cytology, cervical biopsies, or LEEP within 10 months were considered nonadherent and were excluded from the analyses.

Furthermore, some women undergoing active surveillance had a delayed LEEP performed due to progression or persistent disease.^1^ Thus, we divided the active surveillance group into 2 groups: women who had a delayed LEEP performed within 28 months after CIN2 diagnosis and women who had no record (model 2). The window of 28 months was chosen to include results on all LEEPs performed due to progression or persistent disease at 24 months.

### Outcome

The primary outcome was preterm birth (<37 + 0 weeks), while moderately preterm birth (<34 + 0 weeks), extremely preterm birth (<28 + 0 weeks), and PPROM (preterm premature rupture of membranes <37 + 0 weeks) were secondary outcomes. Information on gestational age at birth was obtained from the Danish Medical Birth Registry. During the study period, the term date and, thus, gestational age were mainly derived from ultrasonography scans performed as part of routine antenatal care in the first trimester and were otherwise calculated from the date of the first day in the last menstrual period.^17^ Information on PPROM was collected from the Danish Medical Birth Registry and the Danish National Patient Registry (*International Statistical Classification of Diseases and Related Health Problems, Tenth Revision *[*ICD-10*] codes DO420, DO422, and DO424). To ensure correct linkage of the diagnoses in the Danish National Patient Registry to the included birth, these diagnoses had to be registered between 200 days before and 60 days after the birth.

### Covariates

We obtained information on age, calendar year, residing region, parity, smoking status, body mass index (BMI), prior preterm birth, index cytology, and repeated LEEP from the Danish Civil Registration System, Medical Birth Registry, and Pathology Registry. Index cytology was defined as the most recent cytology within 6 months before to 7 days past CIN2 diagnosis and was classified as normal, low-grade, high-grade, or other or missing. Of note, we only had information on prior births from 1997 and onwards.

### Statistical Analysis

We tabulated characteristics at CIN2 diagnosis and at birth by exposure. Using modified Poisson regression with robust variances,^20,21^ we estimated the crude relative risk (RR) and adjusted RR (aRR) of preterm birth with the immediate LEEP group as reference. We chose this approach because logistic regression, an often-used approach to binary outcomes, provides an odds ratio, and this may be difficult to interpret and overestimates the risk in the case of nonrare events, such as preterm birth.^22^ In an ancillary analysis, we used the active surveillance group without delayed LEEP as reference to compare with the delayed LEEP group.

We performed adjusted analyses by inverse probability treatment weighting (IPTW) and included the following covariates: age and calendar year at CIN2 diagnosis, index cytology, parity, and smoking during pregnancy. For missing values on parity (1%) and smoking (2%), we grouped nulliparous with missing and nonsmoker with missing. For each model (1 and 2), we calculated separate propensity scores using a binomial logistic regression. In IPTW, the goal is to achieve covariate balance, and thus, we assessed the covariate balance by checking the standardized difference between the exposure groups. To reduce the importance of large weights, we applied weight trimming and trimmed the weights to the 99th percentile.^23^

Analyses were stratified by time between CIN2 diagnosis and birth, age at CIN2 diagnosis, index cytology, calendar year of both CIN2 diagnosis and birth, parity, prior preterm birth (multiparous women), smoking status, and BMI. For women undergoing immediate or delayed LEEP (model 2), we also stratified by time since LEEP and birth and whether a repeated LEEP was performed.

We performed several sensitivity analyses. First, we performed a traditional confounder adjustment using a multivariable regression model that included age and calendar year at CIN2 diagnosis, index cytology, parity, and smoking for model 1. Second, we redefined delayed LEEP to also include LEEPs performed beyond the 28-month active surveillance period. Third, we excluded women with repeated LEEP. Fourth, as we only had information on prior births since 1997, we restricted the stratified analysis on prior preterm births to 2003 to 2018 to ensure complete information on prior births. Finally, to check whether our estimates were a result of model choice, we repeated the calculations using logistic regression, including interaction for the included covariates.

Data management was performed in SAS version 9.4 (SAS Institute), and statistical analyses were performed in Stata version 17.0 (StataCorp). Forest plots were created using R version 4.2.3 (R Foundation for Statistical Computing).

## Results

### Characteristics

We identified 10 537 women with a first-time diagnosis of CIN2 and a subsequent singleton birth. The median (IQR) age for the study cohort was 26 (24-29) years. Of these, 4430 women (42%) underwent active surveillance, and 6107 (58%) were treated with immediate LEEP (Table 1). Women who underwent active surveillance were slightly younger than women treated with immediate LEEP (median [IQR] age, 26 [23-28] years and 27 [24-30] years, respectively). For both groups, most were aged 23 to 29 years at CIN2 diagnosis (3125 [70%] and 3907 [64%], respectively). Nearly all women had an abnormal index cytology, with high-grade being predominant in both groups (2044 [46%] and 3310 [54%], respectively). Most women in the active surveillance group were diagnosed after 2007 and gave birth after 2013, while most women treated with immediate LEEP were diagnosed before 2007 and gave birth in 2007 to 2012. In both groups, most women gave birth within 5 years after CIN2 diagnosis (3610 [81%] and 4808 [79%], respectively). Women who underwent active surveillance were more often nulliparous and nonsmokers than women treated with immediate LEEP. Overall, few had a prior preterm birth (179 [2%]).

**Table 1.** Characteristics of Danish Women With a First-Time Diagnosis of CIN2 and a Subsequent Birth, 1998 to 2018

Characteristic	Women, No. (%)		
	Active surveillance (n = 4430)	Immediate LEEP (n = 6107)	Total (N = 10 537)
Age at CIN2 diagnosis, y			
18-22	486 (11.0)	479 (7.8)	965 (9.2)
23-29	3125 (70.5)	3907 (64.0)	7032 (66.7)
30-40	819 (18.5)	1721 (28.2)	2540 (24.1)
Index cytology at CIN2 diagnosis			
Normal	468 (10.6)	406 (6.6)	874 (8.3)
Low-grade^a^	1609 (36.3)	1938 (31.7)	3547 (33.7)
High-grade^b^	2044 (46.1)	3310 (54.2)	5354 (50.8)
Other or missing	309 (7.0)	453 (7.4)	762 (7.2)
Calendar year of CIN2 diagnosis			
1998-2006	1383 (31.2)	2991 (49.0)	4374 (41.5)
2007-2012	1871 (42.2)	2658 (43.5)	4529 (43.0)
2013-2018	1176 (26.6)	458 (7.5)	1634 (15.5)
Calendar year of birth			
1998-2006	677 (15.3)	1286 (21.1)	1963 (18.6)
2007-2012	1044 (23.6)	2534 (41.5)	3578 (34.0)
2013-2018	2709 (61.1)	2287 (37.4)	4996 (47.4)
Years from CIN2 diagnosis to birth			
0-2	1971 (44.5)	2766 (45.3)	4737 (45.0)
3-5	1639 (37.0)	2042 (33.4)	3681 (34.9)
>5	820 (18.5)	1299 (21.3)	2119 (20.1)
Parity at birth			
Nulliparous	3420 (77.2)	4232 (69.3)	7652 (72.6)
Multiparous	995 (22.5)	1822 (29.8)	2817 (26.7)
Missing	15 (0.3)	53 (0.9)	68 (0.6)
Prior preterm birth	56 (1.3)	123 (2.0)	179 (1.7)
Smoking status at birth			
Nonsmoker	3726 (84.1)	4857 (79.5)	8583 (81.5)
Smoker	626 (14.1)	1124 (18.4)	1750 (16.6)
Missing	78 (1.8)	126 (2.1)	204 (1.9)
BMI at birth			
<18.5	161 (3.6)	203 (3.3)	364 (3.5)
18.5-24.9	2813 (63.5)	3757 (61.5)	6570 (62.4)
25.0-29.9	718 (16.2)	1016 (16.6)	1734 (16.5)
≥30.0	331 (7.5)	482 (7.9)	813 (7.7)
Missing	407 (9.2)	649 (10.6)	1056 (10.0)

Abbreviations: BMI, body mass index (calculated as weight in kilograms divided by height in meters squared); CIN2, cervical intraepithelial neoplasia grade 2; LEEP, loop electrosurgical excision procedure.

^a^ Includes atypical squamous cells of undetermined significance and low-grade squamous intraepithelial lesion.

^b^ Includes atypical squamous cells, cannot exclude high-grade squamous intraepithelial lesion; atypical glandular cells; high-grade squamous intraepithelial lesion; adenocarcinoma in situ; and carcinoma.

After application of IPTW, the comparability between women undergoing active surveillance and immediate LEEP increased substantially as all standardized differences were less than 0.1 (eTable 1 in Supplement 1). Thus, the covariates were considered well-balanced across groups.

### Risk of Preterm Birth

Overall, 869 of 10 537 births (8%) were preterm, 280 (3%) were moderately preterm, and 55 (1%) were extremely preterm (Table 2). When comparing active surveillance to immediate LEEP, we found no difference in the risk of preterm birth (aRR, 1.03; 95% CI, 0.90-1.18), moderately preterm birth (aRR, 0.99; 95% CI, 0.77-1.28), or extremely preterm birth (aRR, 0.63; 95% CI, 0.35-1.14). A similar result was seen in the sensitivity analysis with traditional confounder adjustment (eTable 2 in Supplement 1).

**Table 2.** Risk of Preterm, Moderately Preterm, and Extremely Preterm Birth for Women With CIN2 in Denmark, 1998 to 2018

Group	Births, No.	Preterm (<37 + 0 weeks)			Moderately preterm (<34 + 0)			Extremely preterm (<28 + 0)		
		No. (%)	RR (95% CI)	aRR (95% CI)^a^	No. (%)	RR (95% CI)	aRR (95% CI)^a^	No. (%)	RR (95% CI)	aRR (95% CI)^a^
Model 1										
Overall	10 537	869 (8.2)	NA	NA	280 (2.7)	NA	NA	55 (0.5)	NA	NA
Active surveillance	4430	368 (8.3)	1.01 (0.89-1.15)	1.03 (0.90-1.18)	114 (2.6)	0.95 (0.75-1.20)	0.99 (0.77-1.28)	19 (0.4)	0.73 (0.42-1.27)	0.63 (0.35-1.14)
Immediate LEEP	6107	501 (8.2)	1 [Reference]	1 [Reference]	166 (2.7)	1 [Reference]	1 [Reference]	36 (0.6)	1 [Reference]	1 [Reference]
Model 2^b^										
Active surveillance										
No LEEP	2891	202 (7.0)	0.85 (0.73-1.00)	0.88 (0.74-1.04)	60 (2.1)	0.76 (0.57-1.02)	0.80 (0.58-1.10)	7 (0.2)	0.41 (0.18-0.92)	0.28 (0.12-0.65)
Delayed LEEP	1539	166 (10.8)	1.31 (1.11-1.55)	1.29 (1.08-1.55)	54 (3.5)	1.29 (0.95-1.74)	1.30 (0.94-1.81)	12 (0.8)	1.32 (0.69-2.54)	1.16 (0.57-2.36)
Immediate LEEP	6107	501 (8.2)	1 [Reference]	1 [Reference]	166 (2.7)	1 [Reference]	1 [Reference]	36 (0.6)	1 [Reference]	1 [Reference]

Abbreviations: aRR, adjusted relative risk; CIN2, cervical intraepithelial neoplasia grade 2; LEEP, loop electrosurgical excision procedure; NA, not applicable; RR, relative risk.

^a^ aRRs were adjusted for age at CIN2 diagnosis, parity, calendar year at CIN2 diagnosis, index cytology, and smoking status at birth.

^b^ Active surveillance was divided in 2 groups (ie, no LEEP and delayed LEEP), depending on whether a LEEP was performed within 28 months after CIN2 diagnosis.

**Figure 1.**
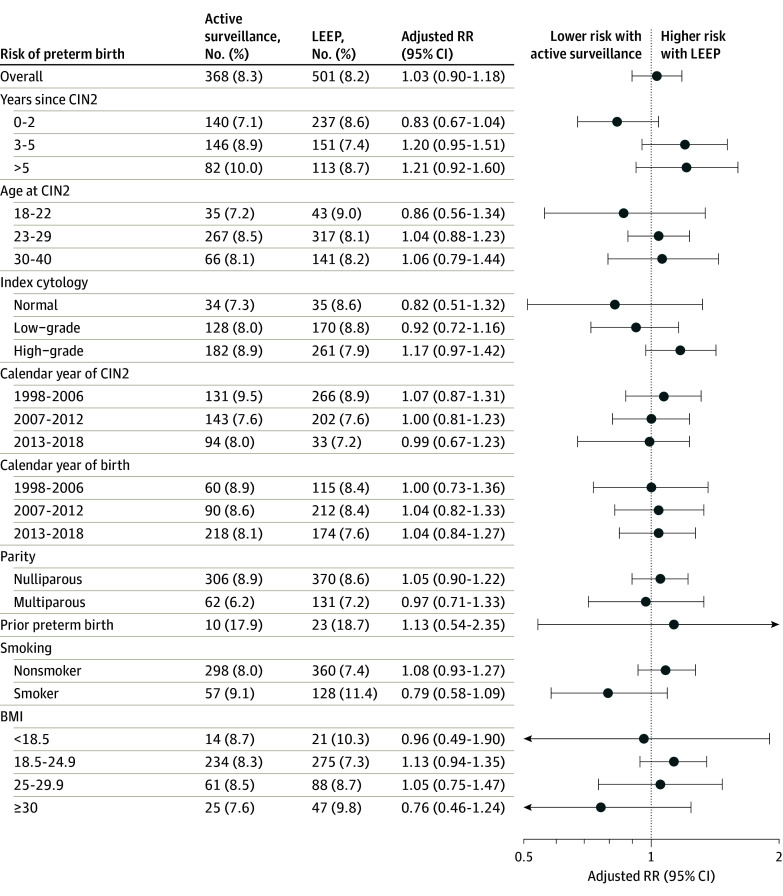
Risk of Preterm Birth for Women With Cervical Intraepithelial Neoplasia Grade 2 (CIN2) Undergoing Either Active Surveillance or Immediate Loop Electrosurgical Excision Procedure (LEEP) Preterm defined as birth at less than 37 + 0 weeks. Relative risk (RR) adjusted for age at CIN2 diagnosis, parity, calendar time at CIN2 diagnosis, index cytology, and smoking status at birth. Body mass index (BMI) was calculated as weight in kilograms divided by height in meters squared.

For women with high-grade index cytology, the risk was slightly increased in women undergoing active surveillance compared with women having an immediate LEEP, but it was not statistically significant (1.17; 95% CI, 0.97-1.42) (Figure 1). The absolute risk of preterm birth was higher in women with a prior preterm birth compared with women without, but the risk was comparable between groups. When restricting to women diagnosed with CIN2 in 2003 to 2018 in a sensitivity analysis, we found similar results (aRR, 1.10; 95% CI, 0.49-2.48). We found no difference in risk of preterm birth across groups in the remaining stratified analyses (Figure 1). Also, when using logistic regression, the estimates were similar (eTable 3 in Supplement 1).

We observed similar findings for moderately preterm birth (eTable 4 in Supplement 1). For the extremely preterm birth, we identified 55 births (Table 2). Due to the low number, interpretation of the effect estimate is difficult, and thus, we were unable to perform stratified analyses for this outcome.

### Active Surveillance With Delayed LEEP

Of women undergoing active surveillance, 1539 (35%) had a delayed LEEP within 28 months. Of these, most were performed within 1 year after CIN2 diagnosis (1210 [79%]). Women with a delayed LEEP were more likely to have a high-grade index cytology (53% vs 42%) but were otherwise comparable with women without a delayed LEEP (eTable 5 in Supplement 1).

**Figure 2.**
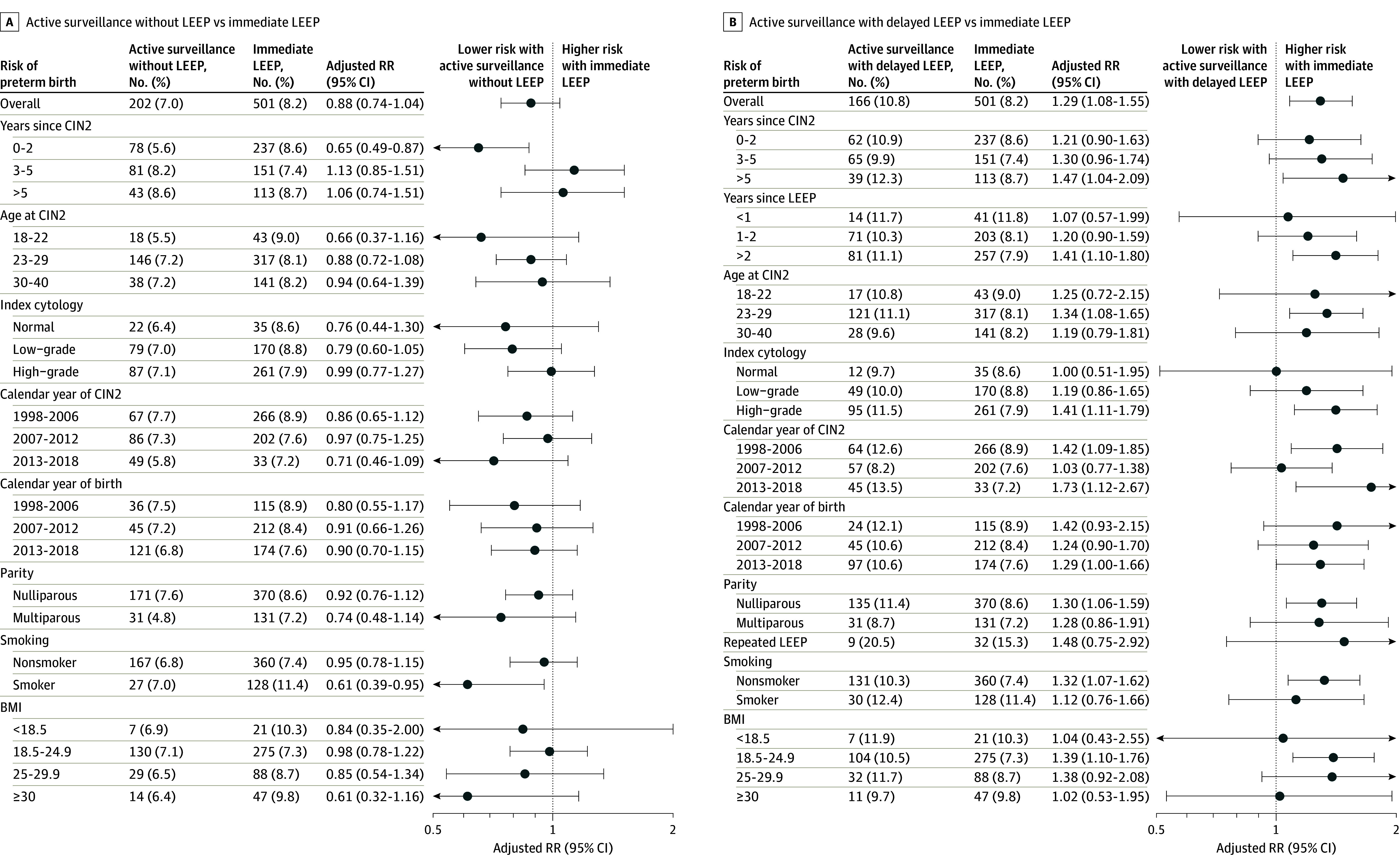
Risk of Preterm Birth Comparing Women With Cervical Intraepithelial Neoplasia Grade 2 (CIN2) Receiving Active Surveillance, Immediate Loop Electrosurgical Excision Procedure (LEEP), and Delayed LEEP Preterm defined as birth at less than 37 + 0 weeks. Relative risks (RR) adjusted for age at CIN2 diagnosis, parity, calendar time at CIN2 diagnosis, index cytology, and smoking status at birth. Body mass index (BMI) was calculated as weight in kilograms divided by height in meters squared.

Compared with immediate LEEP, women undergoing active surveillance without a delayed LEEP had a slightly lower risk of preterm birth (aRR, 0.88; 95% CI, 0.74-1.04) (Table 2). Similar findings were observed in the stratified analyses (Figure 2A). In contrast, the risk of preterm birth was nearly 30% higher in women with delayed LEEP compared with immediate LEEP (aRR, 1.29; 95% CI, 1.08-1.55) (Table 2). In particular, the risk was higher for women who gave birth more than 5 years after CIN2 diagnosis or more than 2 years after LEEP, had a high-grade index cytology, had a repeated LEEP, or were diagnosed with CIN2 between 2013 and 2018 (Figure 2B). Moreover, the time from LEEP to birth was comparable between women treated with delayed (median [IQR], 34.5 [20.0-58.1] months) or immediate LEEP (median [IQR], 38.3 [22.1-64.8] months). We found similar results when delayed LEEP also included LEEPs performed beyond the 28-month surveillance period and when we excluded 254 women with repeated LEEP (eTable 6 and eTable 7 in Supplement 1). Similarly, the risk was approximately 50% higher for women treated with delayed LEEP compared with women undergoing active surveillance without LEEP (eTable 8 in Supplement 1).

### Risk of PPROM

PPROM occurred in 4% of all births (370 of 10 537), with no difference in risk between groups (aRR, 0.91; 95% CI, 0.73-1.14) (eTable 9 in Supplement 1). However, when subdividing active surveillance, the risk of PPROM was nearly 30% lower for women undergoing active surveillance without a delayed LEEP than women with an immediate LEEP (aRR, 0.71; 95% CI, 0.53-0.95). Women who had delayed LEEP had a nearly 30% higher risk of PPROM compared with those who had immediate LEEP, but the results were not statistically significant (aRR, 1.28; 95% CI, 0.97-1.69).

## Discussion

In this study of 10 537 Danish women with CIN2 and a subsequent singleton birth, the absolute risk of preterm birth was 8%. When comparing active surveillance with immediate LEEP, we found a comparable risk of preterm birth. However, for women with a delayed LEEP, the risk of preterm birth was 30% higher compared with women with immediate LEEP. These findings emphasize the need for early identification of women with CIN2 who are at increased risk of progression or persistent CIN2.

Our finding of an 8% absolute risk of preterm birth for women with CIN2 is higher than the risk in the general population, for whom the risk is approximately 5%.^5,6,14^ The finding of a higher risk among women with CIN is consistent with previous studies.^4,5,6,7,11,13,24^ However, the risk of preterm birth depends on whether CIN has been treated or not, ranging from 8% to 10% in women with prior LEEP,^5,6,7^ while it is approximately 7% in women with untreated CIN. This is in line with our study, where we found a nonstatistically significant lower risk (aRR, 0.88; 95% CI, 0.74-1.04) in women with untreated CIN2 (ie, active surveillance without delayed LEEP) compared with women with an immediate LEEP. Yet, our estimate indicates a smaller difference in risk of preterm birth than prior studies.^12,14,25^However, the untreated group in these prior studies may consist of women undergoing colposcopy with or without biopsies and may thus include women without CIN.^12,25^ Thus, the disease severity in these studies is lower than ours given that we solely included women with biopsy-confirmed CIN2.

We found that the risk of preterm birth was 30% higher in women treated with delayed LEEP than immediate LEEP. As studies have shown that most women experience progression of CIN2 instead of persistence,^1,3,26^ disease severity is more likely to be worse in women undergoing delayed LEEP than in women with immediate LEEP.^27^ Increasing disease severity is associated with increasing depth of LEEP.^28^ Hence, the observed increased risk of preterm birth may be explained by a larger volume and/or depth of the cone specimen, which is correlated with the risk of preterm birth.^6,14,28,29^ Unfortunately, we had no information on the cone depth or volume.^29^

Also, the procedure type is associated with preterm birth risk. Cold knife conization is associated with a nearly 3-fold increased risk of preterm birth compared with women in the general population. Among women undergoing LEEP, the risk increase is comparably small—50% to 60%—but still substantial.^6^ Unfortunately, we did not have information on the type of procedure, but LEEP has been increasing in Denmark since the beginning of the 1990s, while use of cold knife conization was practically nonexistent in the study period.^30,31^

Our results highlight the importance of identifying women at CIN2 diagnosis who have a high risk of persistent CIN2 or progression to CIN3+, thus requiring a delayed LEEP. Our results show that a delayed LEEP was associated with a 30% higher risk of preterm birth and PPROM than immediate LEEP. Correspondingly, women without a LEEP during active surveillance had a 12% to 29% lower risk. Risk stratification of women with CIN2 could enable shared decision-making about treatment options for CIN2. Several factors may be considered for risk stratification of CIN2, eg, index cytology and human papillomavirus (HPV) genotype.

We found that women with a high-grade index cytology were more likely to deliver preterm, which is consistent with previous findings of a higher risk of progressing CIN2 among women with high-grade index cytology.^32^ Also, having CIN2 and HPV16 is associated with an increased risk of progression, and women with HPV16 have a higher risk of delivering preterm.^1,2,32,33^

### Limitations

This study also has some limitations. First, CIN2 diagnoses have low reproducibility.^34,35^ However, we expect that potential misclassification is unrelated to whether the women underwent active surveillance, immediate LEEP, or delivered preterm. Second, we lacked information on other factors potentially affecting the risk of preterm birth, eg, socioeconomic status. A lower socioeconomic status is associated with a higher risk of preterm birth,^36,37^ but it is currently unknown whether socioeconomic status is associated with active surveillance of CIN2; however, we note that all women in Denmark have access to free health care. Instead, we had information on and adjusted for smoking during pregnancy, which is correlated with socioeconomic status.^38^ Cervical insufficiency is also a risk factor for preterm birth,^39^ a factor that we unfortunately had no information on. However, women with prior LEEP are more likely to be diagnosed with cervical insufficiency as they are offered ultrasonography of the cervix in gestational age 18 to 20 weeks, while women without LEEP only undergo ultrasonography of the cervix by indication.^40^ Third, we were limited by a low number of events regarding extremely preterm births and were therefore unable to perform stratified analyses on this outcome.

## Conclusions

In this study of women with CIN2 and a subsequent birth, we found no difference in the risk of preterm birth when comparing active surveillance with immediate LEEP. However, the risk of preterm birth and PPROM was 30% higher in women with a delayed LEEP compared with women with an immediate LEEP. These findings highlight the importance of risk stratification at CIN2 diagnosis to ensure identification of women with high risk of progression or persistent CIN2 who may benefit from an immediate LEEP.

## Notes

Supplement 1. **eFigure.** Flowchart of Study Population **eTable 1.** Assessment of Covariate Balance in the Crude Cohort and in the Propensity Score–Weighted Cohort **eTable 2.** Sensitivity Analysis With Traditional Confounder Adjustment (ie, Multivariable Regression Analysis) **eTable 3.** Odds Ratios of Preterm Birth (<37+0 Weeks) in Women With CIN2 Undergoing Active Surveillance or Immediate LEEP Using Logistic Regression (Model 1) **eTable 4.** Risk of Preterm (<37+0 Weeks) and Moderately Preterm Birth (<34+0 Weeks) in Women With CIN2 Undergoing Active Surveillance or Immediate LEEP (Model 1) **eTable 5.** Descriptive Characteristics According to Exposure Status (Model 2) **eTable 6.** Sensitivity Analysis of Delayed LEEP **eTable 7.** Sensitivity Analysis of Repeated LEEP **eTable 8.** Comparison of the Preterm Birth Risk in Women Having No LEEP or Delayed LEEP During Active Surveillance **eTable 9.** The Risk of PPROM in Women With CIN2 Who Underwent Active Surveillance or Immediate LEEP

Supplement 2.
Data Sharing Statement
