# Supplementary material for: Preterm Birth Following Active Surveillance vs Loop Excision for Cervical Intraepithelial Neoplasia Grade 2
Source: JAMA Netw Open. 2024 Mar 14;7(3):e242309. doi: 10.1001/jamanetworkopen.2024.2309 (PMC10940954; doi:10.1001/jamanetworkopen.2024.2309)
Supplement: Supplement 1. — eFigure. Flowchart of Study Population eTable 1. Assessment of Covariate Balance in the Crude Cohort and in the Propensity Score–Weighted Cohort eTable 2. Sensitivity Analysis With Traditional Confounder Adjustment (ie, Multivariable Regression Analysis) eTable 3. Odds Ratios of Preterm Birth (<37+0 Weeks) in Women With CIN2 Undergoing Active Surveillance or Immediate LEEP Using Logistic Regression (Model 1) eTable 4. Risk of Preterm (<37+0 Weeks) and Moderately Preterm Birth (<34+0 Weeks) in Women With CIN2 Undergoing Active Surveillance or Immediate LEEP (Model 1) eTable 5. Descriptive Characteristics According to Exposure Status (Model 2) eTable 6. Sensitivity Analysis of Delayed LEEP eTable 7. Sensitivity Analysis of Repeated LEEP eTable 8. Comparison of the Preterm Birth Risk in Women Having No LEEP or Delayed LEEP During Active Surveillance eTable 9. The Risk of PPROM in Women With CIN2 Who Underwent Active Surveillance or Immediate LEEP [file jamanetwopen-e242309-s001.pdf]

Supplemental Online Content

Lycke KD, Kahlert J, Eriksen DO, et al. Preterm birth following active surveillance vs loop excision for cervical intraepithelial neoplasia grade 2. *JAMA Netw Open.* 2024;7(3):e242309. doi:10.1001/jamanetworkopen.2024.2309

eFigure. Flowchart of Study Population

eTable 1. Assessment of Covariate Balance in the Crude Cohort and in the Propensity Score–Weighted Cohort

eTable 2. Sensitivity Analysis With Traditional Confounder Adjustment (ie, Multivariable Regression Analysis)

eTable 3. Odds Ratios of Preterm Birth (<37+0 Weeks) in Women With CIN2 Undergoing Active Surveillance or Immediate LEEP Using Logistic Regression (Model 1)

eTable 4. Risk of Preterm (<37+0 Weeks) and Moderately Preterm Birth (<34+0 Weeks) in Women With CIN2 Undergoing Active Surveillance or Immediate LEEP (Model 1)

eTable 5. Descriptive Characteristics According to Exposure Status (Model 2)

eTable 6. Sensitivity Analysis of Delayed LEEP

eTable 7. Sensitivity Analysis of Repeated LEEP

eTable 8. Comparison of the Preterm Birth Risk in Women Having No LEEP or Delayed LEEP During Active Surveillance

eTable 9. The Risk of PPROM in Women With CIN2 Who Underwent Active Surveillance or Immediate LEEP

This supplemental material has been provided by the authors to give readers additional information about their work.

© 2024 Lycke KD et al. *JAMA Network Open.*

eFigure. Flowchart of Study Population

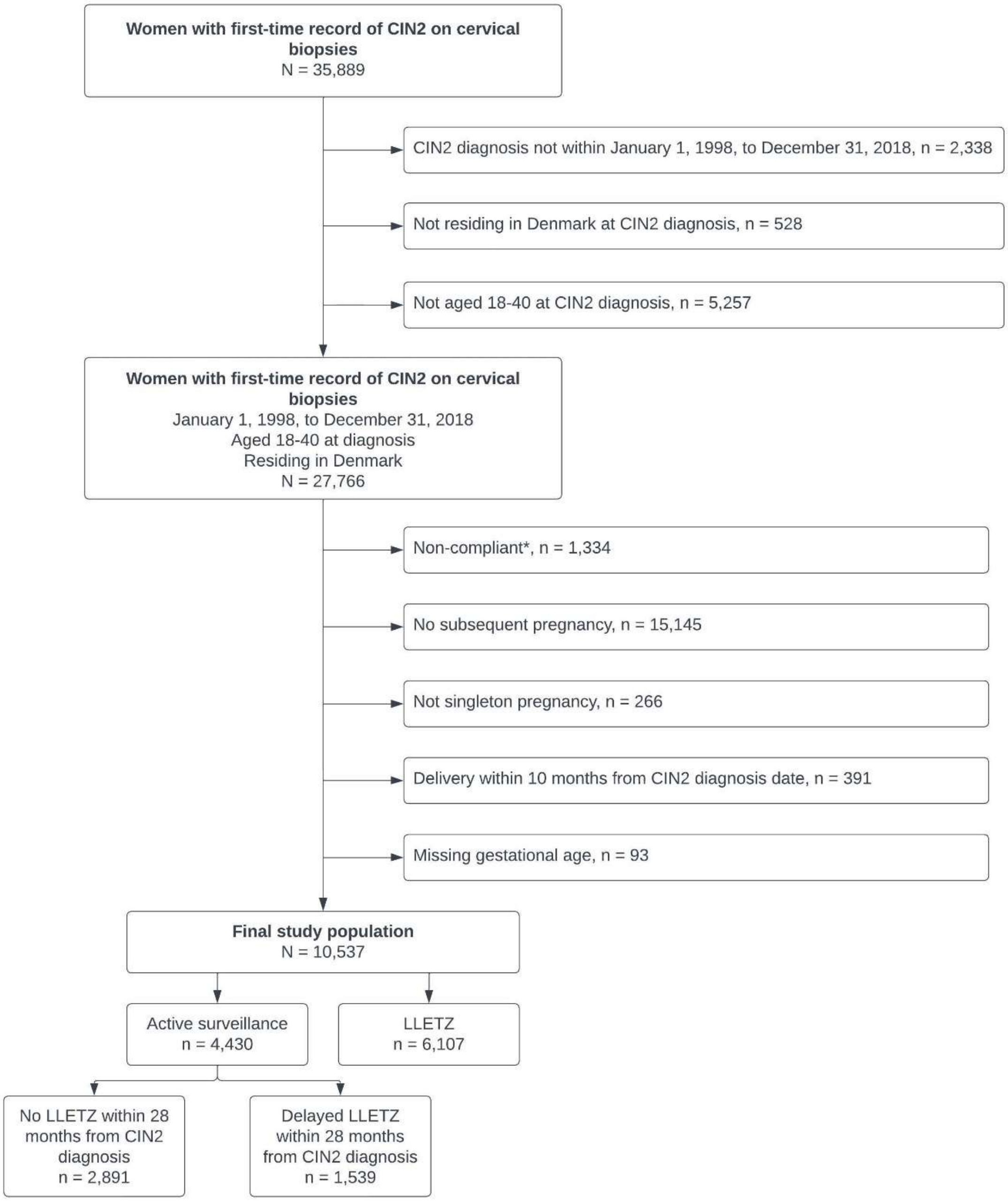

* Non-compliant: No record of cytology, cervical biopsy, or LEEP in the Danish Pathology Registry within 10 months from CIN2 diagnosis.

© 2024 Lycke KD et al. *JAMA Network Open.*

eTable 1. Assessment of Covariate Balance in the Crude Cohort and in the Propensity Score–Weighted Cohort

	Overall crude cohort			Propensity score-weighted cohort		
	**Active surveillance, %**	**LEEP, %**	**SD**	**Active surveillance, %**	**LEEP, %**	**SD**
**Age group**						
18-22	11.0	7.8	0.11	8.8	9.4	0.02
23-29	70.5	64.0	0.14	66.5	65.9	0.01
30-40	18.5	28.2	0.23	24.6	24.7	0.00
**Index cytology**						
Normal	10.6	6.6	0.14	8.2	8.0	0.01
Low-grade[a]	36.3	31.7	0.10	34.3	34.0	0.01
High-grade[b]	46.1	54.2	0.16	51.0	51.4	0.01
Other/missing	7.0	7.4	0.02	7.0	7.0	0.00
**Year of diagnosis**						
1998-2006	31.2	49.0	0.37	42.3	42.1	0.00
2007-2012	42.2	43.5	0.03	42.3	43.2	0.02
2013-2020	26.5	7.5	0.52	15.4	14.6	0.02
**Parity**						
Nulliparous (incl. Missing)	77.5	70.2	0.17	72.1	72.6	0.01
Multiparous	22.5	29.8	0.17	27.9	27.4	0.01
**Smoking**						
Non-smoker (incl. Missing)	84.1	79.5	0.12	83.1	83.2	0.00
Smoker	14.1	18.4	0.12	18.9	16.8	0.00

[a] Includes ASCUS and LSIL

[b] Includes ASC-H, AGC, HSIL, AIS, and carcinoma

© 2024 Lycke KD et al. *JAMA Network Open.*

eTable 2. Sensitivity Analysis With Traditional Confounder Adjustment (ie, Multivariable Regression Analysis)

		Preterm (<37+0)		
	Births, n	n (%)	RR (95% CI)	aRR* (95% CI)
**Model 1**	10,537 (100)	869 (8.2)		
Active Surveillance	4,430 (100)	368 (8.3)	1.01 (0.89-1.15)	1.04 (0.91-1.19)
LEEP	6,107 (100)	501 (8.2)	1.0 (ref.)	1.0 (ref.)
**Model 2**[a]				
Active Surveillance				
No LEEP	2,891 (100)	202 (7.0)	0.85 (0.73-1.00)	0.87 (0.74-1.03)
Delayed LEEP	1,539 (100)	166 (10.8)	1.31 (1.11-1.55)	1.34 (1.13-1.58)
Immediate LEEP	6,107 (100)	501 (8.2)	1.0 (ref.)	1.0 (ref.)

* Adjusted by multivariable regression analysis including age and calendar year at CIN2 diagnosis, index cytology, parity, and smoking

© 2024 Lycke KD et al. *JAMA Network Open.*

eTable 3. Odds Ratios of Preterm Birth (<37+0 Weeks) in Women With CIN2 Undergoing Active Surveillance or Immediate LEEP Using Logistic Regression (Model 1)

		Preterm birth (<37+0)	
	Births, n	n (%)	aOR*
**Time since CIN2**			
**0-2 years**	4,737	377 (8.0)	
Active surveillance	1,971	140 (7.1)	0.82 (0.65-1.05)
LEEP	2,766	237 (8.6)	1.0 (ref.)
**3-5 years**	3,681	297 (8.1)	
Active surveillance	1,639	146 (8.9)	1.21 (0.94-1.56)
LEEP	2,042	151 (7.4)	1.0 (ref.)
**>5 years**	2,119	195 (9.2)	
Active surveillance	820	82 (10.0)	1.24 (0.91-1.68)
LEEP	1,299	113 (8.7)	1.0 (ref.)
**Age at CIN2**			
**18-22**	965	78 (8.1)	
Active surveillance	486	35 (7.2)	0.85 (0.53-1.37)
LEEP	479	43 (9.0)	1.0 (ref.)
**23-29**	7,032	584 (8.3)	
Active surveillance	3,125	267 (8.5)	1.04 (0.87-1.25)
LEEP	3,907	317 (8.1)	1.0 (ref.)
**30-40**	2,540	207 (8.2)	
Active surveillance	819	66 (8.1)	1.07 (0.77-1.49)
LEEP	1,721	141 (8.2)	1.0 (ref.)
**Index cytology**			
**Normal**	874	69 (7.9)	
Active surveillance	468	34 (7.3)	0.81 (0.48-1.35)
LEEP	406	35 (8.6)	1.0 (ref.)
**Low-grade**	3,547	298 (8.4)	
Active surveillance	1,609	128 (8.0)	0.91 (0.70-1.18)
LEEP	1,938	170 (8.8)	1.0 (ref.)
**High-grade**	5,354	443 (8.3)	
Active surveillance	2,044	182 (8.9)	1.19 (0.96-1.47)
LEEP	3,310	261 (7.9)	1.0 (ref.)
**Other/missing**	762	59 (7.7)	
Active surveillance	309	24 (7.8)	0.90 (0.51-1.59)
LEEP	453	35 (7.7)	1.0 (ref.)
**Calendar year of CIN2**			
**1998-2006**	4,374	397 (9.1)	
Active surveillance	1,383	131 (9.5)	1.07 (0.85-1.34)
LEEP	2,991	266 (8.9)	1.0 (ref.)
**2007-2012**	4,529	345 (7.6)	
Active surveillance	1,871	143 (7.6)	1.00 (0.79-1.25)
LEEP	2,658	202 (7.6)	1.0 (ref.)
**2013-2018**	1,634	127 (7.8)	
Active surveillance	1,176	94 (8.0)	0.99 (0.65-1.51)
LEEP	458	33 (7.2)	1.0 (ref.)
**Calendar year of birth**			

© 2024 Lycke KD et al. *JAMA Network Open.*

**1998-2006**	1,963	175 (8.9)	
Active surveillance	677	60 (8.9)	1.00 (0.71-1.40)
LEEP	1,286	115 (8.9)	1.0 (ref.)
**2007-2012**	3,578	302 (8.4)	
Active surveillance	1,044	90 (8.6)	1.05 (0.80-1.36)
LEEP	2,534	212 (8.4)	1.0 (ref.)
**2013-2018**	4,996	392 (7.9)	
Active surveillance	2,709	218 (8.1)	1.04 (0.83-1.30)
LEEP	2,287	174 (7.6)	1.0 (ref.)
**Parity**			
**Nulliparous**	7,720	676 (8.8)	
Active surveillance	3,435	306 (8.9)	1.05 (0.89-1.25)
LEEP	4,285	370 (8.6)	1.0 (ref.)
**Multiparous**	2,817	193 (6.9)	
Active surveillance	995	62 (6.2)	0.96 (0.69-1.35)
LEEP	1,822	131 (7.2)	1.0 (ref.)
**Prior preterm birth**	179	33 (18.4)	
Active surveillance	56	10 (17.9)	1.16 (0.47-2.86)
LEEP	123	23 (18.7)	1.0 (ref.)
**Smoking (birth)**			
**Non-smoker**	8,583	658 (7.7)	
Active surveillance	3,726	298 (8.0)	1.09 (0.92-1.30)
LEEP	4,857	360 (7.4)	1.0 (ref.)
**Smoker**	1,750	185 (10.6)	
Active surveillance	626	57 (9.1)	0.77 (0.54-1.10)
LEEP	1,124	128 (11.4)	1.0 (ref.)
**BMI (birth)**			
**<18.5**	364	35 (9.6)	
Active surveillance	161	14 (8.7)	0.96 (0.45-2.02)
LEEP	203	21 (10.3)	1.0 (ref.)
**18.5-24.9**	6,570	509 (7.8)	
Active surveillance	2,813	234 (8.3)	1.14 (0.94-1.39)
LEEP	3,757	275 (7.3)	1.0 (ref.)
**25-29.9**	1,734	149 (8.6)	
Active surveillance	718	61 (8.5)	1.06 (0.73-1.53)
LEEP	1,016	88 (8.7)	1.0 (ref.)
**≥30**	813	72 (8.9)	
Active surveillance	331	25 (7.6)	0.74 (0.43-1.26)
LEEP	482	47 (9.8)	1.0 (ref.)

* Adjusted by age and calendar year at CIN2 diagnosis, index cytology, parity, and smoking, including interaction
Abbreviations: aOR: adjusted odds ratio

© 2024 Lycke KD et al. *JAMA Network Open.*

eTable 4. Risk of Preterm (<37+0 Weeks) and Moderately Preterm Birth (<34+0 Weeks) in Women With CIN2 Undergoing Active Surveillance or Immediate LEEP (Model 1)

		Preterm (<37+0)			Moderately preterm (<34+0)		
	Births, n	n (%)	RR	aRR*	n (%)	RR	aRR*
**Time since CIN2**							
**0-2 years**	4,737	377 (8.0)			119 (2.5)		
Active surveillance	1,971	140 (7.1)	0.83 (0.68-1.01)	0.83 (0.67-1.04)	45 (2.3)	0.85 (0.59-1.23)	0.83 (0.56-1.23)
LEEP	2,766	237 (8.6)	1.0 (ref.)	1.0 (ref.)	74 (2.7)	1.0 (ref.)	1.0 (ref.)
**3-5 years**	3,681	297 (8.1)			94 (2.6)		
Active surveillance	1,639	146 (8.9)	1.20 (0.97-1.50)	1.20 (0.95-1.51)	42 (2.6)	1.01 (0.67-1.50)	1.08 (0.70-1.67)
LEEP	2,042	151 (7.4)	1.0 (ref.)	1.0 (ref.)	52 (2.6)	1.0 (ref.)	1.0 (ref.)
**>5 years**	2,119	195 (9.2)			67 (3.2)		
Active surveillance	820	82 (10.0)	1.15 (0.88-1.51)	1.21 (0.92-1.60)	27 (3.3)	1.07 (0.66-1.73)	1.18 (0.72-1.93)
LEEP	1,299	113 (8.7)	1.0 (ref.)	1.0 (ref.)	40 (3.1)	1.0 (ref.)	1.0 (ref.)
**Age at CIN2**							
**18-22**	965	78 (8.1)			23 (2.4)		
Active surveillance	486	35 (7.2)	0.80 (0.52-1.23)	0.86 (0.56-1.34)	11 (2.3)	0.90 (0.40-2.03)	1.00 (0.44-2.30)
LEEP	479	43 (9.0)	1.0 (ref.)	1.0 (ref.)	12 (2.5)	1.0 (ref.)	1.0 (ref.)
**23-29**	7,032	584 (8.3)			177 (2.5)		
Active surveillance	3,125	267 (8.5)	1.05 (0.90-1.23)	1.04 (0.88-1.23)	83 (2.7)	1.10 (0.82-1.48)	1.15 (0.85-1.57)
LEEP	3,907	317 (8.1)	1.0 (ref.)	1.0 (ref.)	94 (2.4)	1.0 (ref.)	1.0 (ref.)
**30-40**	2,540	207 (8.2)			80 (3.2)		
Active surveillance	819	66 (8.1)	0.98 (0.74-1.30)	1.06 (0.79-1.44)	20 (2.4)	0.70 (0.43-1.15)	0.69 (0.40-1.21)
LEEP	1,721	141 (8.2)	1.0 (ref.)	1.0 (ref.)	60 (3.5)	1.0 (ref.)	1.0 (ref.)
**Index cytology**							
**Normal**	874	69 (7.9)			17 (2.0)		
Active surveillance	468	34 (7.3)	0.84 (0.54-1.33)	0.82 (0.51-1.32)	8 (1.7)	0.77 (0.30-1.98)	0.84 (0.31-2.24)
LEEP	406	35 (8.6)	1.0 (ref.)	1.0 (ref.)	9 (2.2)	1.0 (ref.)	1.0 (ref.)
**Low-grade**	3,547	298 (8.4)			95 (2.7)		
Active surveillance	1,609	128 (8.0)	0.91 (0.73-1.13)	0.92 (0.72-1.16)	38 (2.4)	0.80 (0.54-1.20)	0.90 (0.58-1.39)
LEEP	1,938	170 (8.8)	1.0 (ref.)	1.0 (ref.)	57 (2.9)	1.0 (ref.)	1.0 (ref.)
**High-grade**	5,354	443 (8.3)			146 (2.7)		
Active surveillance	2,044	182 (8.9)	1.13 (0.94-1.35)	1.17 (0.97-1.42)	59 (2.9)	1.10 (0.79-1.52)	1.12 (0.79-1.58)
LEEP	3,310	261 (7.9)	1.0 (ref.)	1.0 (ref.)	87 (2.6)	1.0 (ref.)	1.0 (ref.)
**Other/missing**	762	59 (7.7)			22 (2.9)		
Active surveillance	309	24 (7.8)	1.01 (0.61-1.66)	0.91 (0.54-1.54)	9 (2.9)	1.01 (0.44-2.35)	0.86 (0.35-2.08)
LEEP	453	35 (7.7)	1.0 (ref.)	1.0 (ref.)	13 (2.9)	1.0 (ref.)	1.0 (ref.)

© 2024 Lycke KD et al. *JAMA Network Open.*

		Preterm (<37+0)			Moderately preterm (<34+0)		
	Births, n	n (%)	RR	aRR*	n (%)	RR	aRR*
**Calendar year of CIN2**							
**1998-2006**	4,374	397 (9.1)			132 (3.0)		
Active surveillance	1,383	131 (9.5)	1.07 (0.87-1.30)	1.07 (0.87-1.31)	46 (3.3)	1.16 (0.81-1.64)	1.16 (0.81-1.66)
LEEP	2,991	266 (8.9)	1.0 (ref.)	1.0 (ref.)	86 (2.9)	1.0 (ref.)	1.0 (ref.)
**2007-2012**	4,529	345 (7.6)			108 (2.4)		
Active surveillance	1,871	143 (7.6)	1.01 (0.82-1.24)	1.00 (0.81-1.23)	38 (2.0)	0.77 (0.52-1.14)	0.78 (0.52-1.16)
LEEP	2,658	202 (7.6)	1.0 (ref.)	1.0 (ref.)	70 (2.6)	1.0 (ref.)	1.0 (ref.)
**2013-2018**	1,634	127 (7.8)			40 (2.5)		
Active surveillance	1,176	94 (8.0)	1.11 (0.76-1.62)	0.99 (0.67-1.46)	30 (2.6)	1.17 (0.58-2.37)	1.12 (0.54-2.33)
LEEP	458	33 (7.2)	1.0 (ref.)	1.0 (ref.)	10 (2.2)	1.0 (ref.)	1.0 (ref.)
**Calendar year of birth**							
**1998-2006**	1,963	175 (8.9)			51 (2.6)		
Active surveillance	677	60 (8.9)	0.99 (0.74-1.34)	1.00 (0.73-1.36)	20 (3.0)	1.23 (0.70-2.13)	1.28 (0.72-2.27)
LEEP	1,286	115 (8.9)	1.0 (ref.)	1.0 (ref.)	31 (2.4)	1.0 (ref.)	1.0 (ref.)
**2007-2012**	3,578	302 (8.4)			106 (3.0)		
Active surveillance	1,044	90 (8.6)	1.03 (0.81-1.30)	1.04 (0.82-1.33)	29 (2.8)	0.91 (0.60-1.39)	0.92 (0.60-1.41)
LEEP	2,534	212 (8.4)	1.0 (ref.)	1.0 (ref.)	77 (3.0)	1.0 (ref.)	1.0 (ref.)
**2013-2018**	4,996	392 (7.9)			123 (2.5)		
Active surveillance	2,709	218 (8.1)	1.06 (0.87-1.28)	1.04 (0.84-1.27)	65 (2.4)	0.95 (0.67-1.34)	0.97 (0.67-1.41)
LEEP	2,287	174 (7.6)	1.0 (ref.)	1.0 (ref.)	58 (2.5)	1.0 (ref.)	1.0 (ref.)
**Parity**							
**Nulliparous**	7,720	676 (8.8)			219 (2.8)		
Active surveillance	3,435	306 (8.9)	1.03 (0.89-1.19)	1.05 (0.90-1.22)	101 (2.9)	1.07 (0.82-1.39)	1.13 (0.85-1.49)
LEEP	4,285	370 (8.6)	1.0 (ref.)	1.0 (ref.)	118 (2.8)	1.0 (ref.)	1.0 (ref.)
**Multiparous**	2,817	193 (6.9)			61 (2.2)		
Active surveillance	995	62 (6.2)	0.87 (0.65-1.16)	0.97 (0.71-1.33)	13 (1.3)	0.50 (0.27-0.91)	0.61 (0.32-1.18)
LEEP	1,822	131 (7.2)	1.0 (ref.)	1.0 (ref.)	48 (2.6)	1.0 (ref.)	1.0 (ref.)
**Prior preterm birth**	179	33 (18.4)			-	-	-
Active surveillance	56	10 (17.9)	1.02 (0.90-1.17)	1.13 (0.54-2.35)	-	-	-
LEEP	123	23 (18.7)	1.0 (ref.)	1.0 (ref.)	-	-	-

© 2024 Lycke KD et al. *JAMA Network Open.*

		Preterm (<37+0)			Moderately preterm (<34+0)		
	Births, n	n (%)	RR	aRR*	n (%)	RR	aRR*
**Smoking (birth)**							
**Non-smoker**	8,583	658 (7.7)			205 (2.4)		
Active surveillance	3,726	298 (8.0)	1.08 (0.93-1.25)	1.08 (0.93-1.27)	90 (2.4)	1.02 (0.78-1.34)	1.02 (0.76-1.36)
LEEP	4,857	360 (7.4)	1.0 (ref.)	1.0 (ref.)	115 (2.4)	1.0 (ref.)	1.0 (ref.)
**Smoker**	1,750	185 (10.6)			66 (3.8)		
Active surveillance	626	57 (9.1)	0.80 (0.59-1.08)	0.79 (0.58-1.09)	20 (3.2)	0.78 (0.47-1.31)	0.90 (0.52-1.55)
LEEP	1,124	128 (11.4)	1.0 (ref.)	1.0 (ref.)	46 (4.1)	1.0 (ref.)	1.0 (ref.)
**BMI (birth)**							
**<18.5**	364	35 (9.6)			12 (3.3)		
Active surveillance	161	14 (8.7)	0.84 (0.44-1.60)	0.96 (0.49-1.90)	5 (3.1)	0.90 (0.29-2.79)	1.44 (0.45-4.59)
LEEP	203	21 (10.3)	1.0 (ref.)	1.0 (ref.)	7 (3.5)	1.0 (ref.)	1.0 (ref.)
**18.5-24.9**	6,570	509 (7.8)			152 (2.3)		
Active surveillance	2,813	234 (8.3)	1.14 (0.96-1.34)	1.13 (0.94-1.35)	69 (2.5)	1.11 (0.81-1.52)	1.14 (0.82-1.60)
LEEP	3,757	275 (7.3)	1.0 (ref.)	1.0 (ref.)	83 (2.2)	1.0 (ref.)	1.0 (ref.)
**25-29.9**	1,734	149 (8.6)			46 (2.7)		
Active surveillance	718	61 (8.5)	0.98 (0.72-1.34)	1.05 (0.75-1.47)	16 (2.2)	0.75 (0.41-1.37)	0.71 (0.38-1.34)
LEEP	1,016	88 (8.7)	1.0 (ref.)	1.0 (ref.)	30 (3.0)	1.0 (ref.)	1.0 (ref.)
**≥30**	813	72 (8.9)			31 (3.8)		
Active surveillance	331	25 (7.6)	0.77 (0.49-1.23)	0.76 (0.46-1.24)	10 (3.0)	0.69 (0.33-1.45)	0.61 (0.27-1.32)
LEEP	482	47 (9.8)	1.0 (ref.)	1.0 (ref.)	21 (4.4)	1.0 (ref.)	1.0 (ref.)

* Adjusted for age at CIN2 diagnosis, parity, calendar time at CIN2 diagnosis, index cytology, and smoking status

© 2024 Lycke KD et al. *JAMA Network Open.*

eTable 5. Descriptive Characteristics According to Exposure Status (Model 2)

	CIN2			
	Active surveillance		Immediate LEEP	Total
	No LEEP[a]	Delayed LEEP[b]		
	n (%)	n (%)	n (%)	n (%)
**Total**	2,891 (100)	1,539 (100)	6,107 (100)	10,537 (100)
**Age, median (IQR)**	26 (23-28)	26 (23-28)	27 (24-30)	26 (24-29)
**Age group (birth)**				
18-22	328 (11.4)	158 (10.3)	479 (7.8)	965 (9.2)
22-29	2,036 (70.4)	1,089 (70.8)	3,907 (64.0)	7,032 (66.7)
30-40	527 (18.2)	292 (19.0)	1,721 (28.2)	2,540 (24.1)
**Calendar year of CIN2**				
1998-2006	873 (30.2)	510 (33.1)	2,991 (49.0)	4,374 (41.5)
2007-2012	1,175 (40.6)	696 (45.2)	2,658 (43.5)	4,529 (43.0)
2013-2018	843 (29.2)	333 (21.6)	458 (7.5)	1,634 (15.5)
**Calendar year of birth**				
1998-2006	478 (16.5)	199 (12.9)	1,286 (21.1)	1,963 (18.6)
2007-2012	621 (21.5)	423 (27.5)	2,534 (41.5)	3,578 (34.0)
2013-2018	1,792 (62.0)	917 (59.6)	2,287 (37.4)	4,996 (47.4)
**Region**				
Capital	663 (22.9)	375 (24.4)	3,014 (49.4)	4,052 (38.4)
Central	1,417 (49.0)	747 (48.5)	568 (9.3)	2,732 (25.9)
Northern	250 (8.6)	101 (6.6)	769 (12.6)	1,120 (10.6)
Zealand	118 (4.1)	53 (3.4)	547 (9.0)	718 (6.8)
Southern	443 (15.3)	263 (17.1)	1,209 (19.8)	1,915 (18.2)
**Index cytology**				
Normal	344 (11.9)	124 (8.1)	406 (6.6)	874 (8.3)
Low-grade	1,120 (38.7)	489 (31.8)	1,938 (31.7)	3,547 (33.7)
High-grade	1,220 (42.2)	824 (53.5)	3,310 (54.2)	5,354 (50.8)
Other/missing	207 (7.2)	102 (6.6)	453 (7.4)	762 (7.2)
**Time from CIN2 until birth**				
0-2 years	1,404 (48.6)	567 (36.8)	2,766 (45.3)	4,737 (45.0)
3-5 years	984 (34.0)	655 (42.6)	2,042 (33.4)	3,681 (34.9)
>5 years	503 (17.4)	317 (20.6)	1,299 (21.3)	2,119 (20.1)
**Time from LEEP until birth**				
<1 year	-	120 (7.8)	348 (5.7)	-
1-2 years	-	691 (44.9)	2,518 (41.2)	-
>2 years	-	728 (47.3)	3,241 (53.7)	-
**Parity (birth)^2**				
Nulliparous	2,240 (77.5)	1,180 (76.7)	4,232 (69.3)	7,652 (72.6)
**Prior preterm birth**	32 (1.1)	24 (1.6)	123 (2.0)	179 (1.7)
**Repeat LEEP**	-	44 (2.9)	209 (3.4)	-
**Smoking (birth)**				
Non-smoker	2,451 (84.8)	1,275 (82.8)	4,857 (79.5)	8,583 (81.5)
Smoker	384 (13.3)	242 (15.7)	1,124 (18.4)	1,750 (16.6)
Missing	56 (1.9)	22 (1.4)	126 (2.1)	204 (1.9)

© 2024 Lycke KD et al. *JAMA Network Open.*

	CIN2			
	Active surveillance		Immediate LEEP	Total
**BMI (birth)**				
<18.5	102 (3.5)	59 (3.8)	203 (3.3)	364 (3.5)
18.5-24.9	1,824 (63.1)	989 (64.3)	3,757 (61.5)	6,570 (62.4)
25-29.9	445 (15.4)	273 (17.7)	1,016 (16.6)	1,734 (16.5)
≥30	218 (7.5)	113 (7.3)	482 (7.9)	813 (7.7)
Missing	302 (10.5)	105 (6.8)	649 (10.6)	1,056 (10.0)

[a] Defined by whether a LEEP was performed within 28 months from CIN2 diagnosis and prior to birth
[b] Due to low number of events for missing, we were unable to report the numbers for multiparous and missing

© 2024 Lycke KD et al. *JAMA Network Open*.

eTable 6. Sensitivity Analysis of Delayed LEEP

		Preterm births (<37+0)		
	n	n (%)	RR (95% CI)	aRR (95% CI)*
**Model 2 (original)**				
Active surveillance	2,891	202 (7.0)	0.85 (0.73-1.00)	0.88 (0.74-1.04)
Delayed LEEP (within 28 months)	1,539	166 (10.8)	1.31 (1.11-1.55)	1.29 (1.08-1.55)
Immediate LEEP	6,107	501 (8.2)	1.0 (ref.)	1.0 (ref.)
**Model 2 (new definition of delayed LEEP)[a]**				
Active surveillance (no LEEP prior to birth)	2,788	189 (6.8)	0.83 (0.70-0.97)	0.86 (0.72-1.02)
Delayed LEEP (LEEP prior to birth)	1,642	179 (10.9)	1.33 (1.13-1.56)	1.31 (1.10-1.56)
Immediate LEEP	6,107	501 (8.2)	1.0 (ref.)	1.0 (ref.)

[a] includes LEEPs performed beyond the 28-month active surveillance period, but prior to birth

* Adjusted for age at CIN2 diagnosis, parity, calendar time at CIN2 diagnosis, index cytology, and smoking status at birth

© 2024 Lycke KD et al. *JAMA Network Open.*

eTable 7. Sensitivity Analysis of Repeated LEEP

Women with repeated LEEP during follow-up was excluded (n=254)

		Preterm (<37+0)		
	Births, n	n (%)	RR (95% CI)	aRR* (95% CI)
**Model 1**	10,283	828 (8.0)		
Active surveillance	4,385	359 (8.2)	1.03 (0.90-1.17)	1.04 (0.90-1.20)
LEEP	5,898	469 (8.0)	1.0 (ref.)	1.0 (ref.)
**Model 2**[a]				
Active surveillance				
No LEEP	2,890	202 (7.0)	0.88 (0.75-1.03)	0.90 (0.76-1.07)
Delayed LEEP	1,495	157 (10.5)	1.32 (1.11-1.57)	1.29 (1.07-1.55)
Immediate LEEP	5,898	469 (8.0)	1.0 (ref.)	1.0 (ref.)

[a] Active surveillance is subdivided into two depending on whether a subsequent LEEP was performed within 28 months from CIN2 diagnosis

* Adjusted for age at CIN2 diagnosis, parity, calendar time at CIN2 diagnosis, index cytology, and smoking status at birth

© 2024 Lycke KD et al. *JAMA Network Open.*

eTable 8. Comparison of the Preterm Birth Risk in Women Having No LEEP or Delayed LEEP During Active Surveillance

		Preterm (<37+0)		
Active surveillance	Births, n	n (%)	RR (95% CI)	aRR* (95% CI)
No LEEP	2,891 (100)	202 (7.0)	1.0 (ref.)	1.0 (ref.)
Delayed LEEP	1,539 (100)	166 (10.8)	1.54 (1.27-1.88)	1.55 (1.27-1.90)

* Adjusted for age at CIN2 diagnosis, parity, calendar time at CIN2 diagnosis, index cytology, and smoking status at birth

© 2024 Lycke KD et al. *JAMA Network Open.*

eTable 9. The Risk of PPROM in Women With CIN2 Who Underwent Active Surveillance or Immediate LEEP

		PPROM		
	Births, n	n (%)	RR (95% CI)	aRR* (95% CI)
**Model 1**				
Active surveillance	4,430 (100)	149 (3.4)	0.93 (0.76-1.14)	0.91 (0.73-1.14)
LEEP	6,107 (100)	221 (3.6)	1.0 (ref.)	1.0 (ref.)
**Model 2[a]**				
Active surveillance				
No LEEP	2,891 (100)	74 (2.6)	0.71 (0.55-0.92)	0.71 (0.53-0.95)
Delayed LEEP	1,539 (100)	75 (4.9)	1.35 (1.04-1.74)	1.28 (0.97-1.69)
Immediate LEEP	6,107 (100)	221 (3.6)	1.0 (ref.)	1.0 (ref.)

[a] Active surveillance is subdivided into two depending on whether a subsequent LEEP was performed within 28 months from CIN2 diagnosis

* Adjusted for age at CIN2 diagnosis, parity, calendar time at CIN2 diagnosis, index cytology, and smoking status at birth

© 2024 Lycke KD et al. *JAMA Network Open.*
