# Supplementary material for: Preterm Birth Following Active Surveillance vs Loop Excision for Cervical Intraepithelial Neoplasia Grade 2
Source: JAMA Netw Open. 2024 Mar 14;7(3):e242309. doi: 10.1001/jamanetworkopen.2024.2309 (PMC10940954; doi:10.1001/jamanetworkopen.2024.2309)
Supplement: Supplement 2. — Data Sharing Statement [file jamanetwopen-e242309-s002.pdf]

Data Sharing Statement

Lycke. Preterm Birth Following Active Surveillance vs Loop Excision for Cervical Intraepithelial Neoplasia Grade 2. *JAMA Netw Open*. Published March 14, 2024.
doi:10.1001/jamanetworkopen.2024.2309

Data
Data available: No

Additional Information
Explanation for why data not available: This study was performed on the remote servers of the Danish Health Data Authority. Due to restrictions by the Danish Health Data Authority, individual-level data cannot be shared by the authors, and cell counts with less than five cannot be reported. Data and statistical code can be accessed after application to the Danish Health Data Authority. The study protocol is available upon request to the corresponding author up to two years after publication.
